# Associations between metabolic overweight/obesity phenotypes and mortality risk among patients with chronic heart failure

**DOI:** 10.3389/fendo.2024.1445395

**Published:** 2024-09-20

**Authors:** You Zhou, Yingli Xie, Jingjing Dong, Kunlun He

**Affiliations:** ^1^ School of Medicine, Nankai University, Tianjin, China; ^2^ The First Affiliated Hospital, Collage of Clinical Medicine of Henan University of Science and Technology, Luoyang, China; ^3^ Medical Innovation Research Department of People’s Liberation Army General Hospital, Beijing, China

**Keywords:** chronic heart failure, mortality, metabolic syndrome, overweight or obesity, corhort study

## Abstract

**Background:**

Metabolic disorders and overweight or obesity are highly prevalent and intricately linked in patients with chronic heart failure (CHF). However, it remains unclear whether there is an interactive effect between these conditions and the prognosis of heart failure, and whether such an interaction is influenced by stratification based on age and sex.

**Methods:**

A total of 4,955 patients with CHF were enrolled in this study. Metabolic status was assessed according to the presence or absence of metabolic syndrome (MetS). BMI categories included normal weight and overweight or obesity (BMI < 24, ≥ 24 kg/m^2^). Patients were divided into four phenotypes according to their metabolic status and BMI: metabolically healthy with normal weight (MHNW), metabolically unhealthy with normal weight (MUNW), metabolically healthy with overweight or obesity (MHO), and metabolically unhealthy with overweight or obesity (MUO). The incidence of primary outcomes, including all-cause and cardiovascular (CV) death, was recorded.

**Results:**

During a mean follow-up of 3.14 years, a total of 1,388 (28.0%) all-cause deaths and 815 (16.4%) CV deaths were documented. Compared to patients with the MHNW phenotype, those with the MUNW (adjusted hazard ratio [aHR], 1.66; 95% confidence interval [CI], 1.38–2.00) or MUO (aHR, 1.42 [95% CI, 1.24–1.63]) phenotypes had a greater risk of all-cause death, and those with the MHO phenotype (aHR, 0.61 [95% CI, 0.51–0.72]) had a lower risk of all-cause death. Moreover, the above phenomenon existed mainly among males and elderly females (aged ≥ 60 years). In nonelderly females (aged < 60 years), the detrimental effects of MetS were lower (aHR, 1.05 [95% CI, 0.63–1.75] among MUNW group and aHR, 0.52 [95% CI, 0.34–0.80] among MUO group), whereas the protective effects of having overweight or obesity persisted irrespective of metabolic status (aHR, 0.43 [95% CI, 0.26–0.69] among MHO group and aHR, 0.52 [95% CI, 0.34–0.80] among MUO group). Similar results were obtained in the Cox proportional risk analysis of the metabolic overweight/obesity phenotypes and CV death.

**Conclusions:**

In male and elderly female patients with CHF, the detrimental effects of MetS outweighed the protective benefits of having overweight or obesity. Conversely, in nonelderly females, the protective effects of having overweight or obesity were significantly greater than the adverse impacts of MetS.

## Introduction

Cardiovascular disease (CVD) remains a significant global health challenge and is a leading cause of mortality and morbidity. According to statistics, the global incidence of CVD nearly doubled from 1990 to 2019, increasing from 217 million to 523 million cases, with corresponding deaths increasing from 12.1 million to 18.6 million (1). Chronic heart failure (CHF), a heterogeneous syndrome representing the end stage of various CVDs, is a significant contributor to global mortality, affecting 1–2% of adults worldwide (2). Factors such as population aging and increased life expectancy are driving the increasing prevalence of heart failure (HF) (3). CHF imposes a significant strain on health systems due to its high morbidity, high mortality rate, and negative influence on patient quality of life (4). Therefore, conducting an in-depth exploration of the prognostic factors and risk stratification for CHF is crucial to effectively reduce this ongoing burden.

Metabolic syndrome (MetS) encompasses a range of cardiovascular risk factors, including insulin resistance (IR), hypertension, dyslipidemia, and obesity, all of which heighten the risk of HF (5). Studies have shown that MetS is a significant risk factor for the onset and progression of HF and demonstrates a significant prevalence among patients with HF (5, 6). IR is the core feature of MetS, and numerous studies have established that IR significantly correlates with poor prognosis among patients with various CVDs, including HF (7–9). However, there remains controversy regarding the relationship between MetS and the prognosis of patients with HF. While some studies have indicated that MetS is linked to a worse prognosis of patients with HF (10–12), others have demonstrated no such association (13, 14). These discrepancies may stem from differences in the composition of the study populations and their metabolic profiles.

Obesity, as a key component of MetS, has a controversial impact on the prognosis of patients with HF, especially considering the obesity paradox. Some evidence supports the obesity paradox, which suggests that although having overweight or obesity is associated with a higher incidence of chronic diseases, it is closely related to better prognoses (15, 16). However, other studies challenge this idea, questioning its generalizability and applicability to specific groups (17–19).

Although MetS is frequently associated with overweight and obesity, it is important to note that not all individuals with MetS have overweight or obesity. Similarly, not every person with overweight or obesity has MetS; thus, this disease presentation demonstrates intersecting phenotypes. Few studies have specifically investigated the impact of metabolic overweight/obesity phenotypes on mortality outcomes in patients with CHF. Moreover, the complexities of the interaction between obesity and MetS and their impact on the prognosis of patients with HF remain incompletely understood (5). Therefore, this study aimed to bridge this significant knowledge gap by examining the relationship between different metabolic overweight/obesity phenotypes and mortality risk in patients with CHF. Additionally, considering that metabolic levels may be significantly influenced by age and sex (20, 21), we further conducted exploratory analyses according to sex–age stratification.

## Methods

### Study design and population

**Figure 1:**
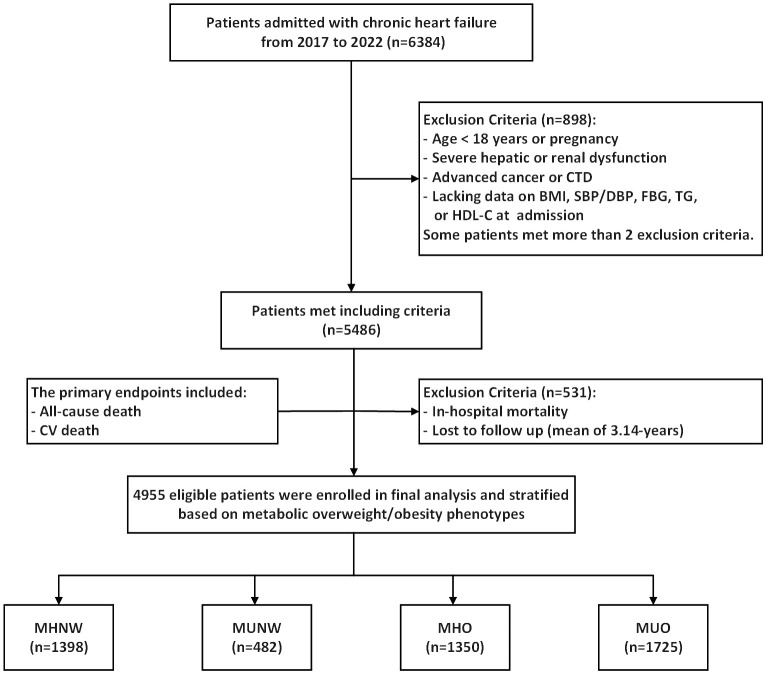
Flow diagram of patients selection. CTD, connective tissue diseases; BMI, body mass Index; SBP, systolic blood pressure; DBP, diastolic blood pressure; FBG, fasting blood glucose; TG, triglyceride; HDL-C, high density lipoprotein cholesterol; CV death, cardiovascular death; MHNW, metabolically healthy with normal weight; MUNW, metabolically unhealthy with normal weight; MHO, metabolically healthy with overweight or obesity; MUO, metabolically unhealthy with overweight or obesity.

In this study, we conducted a retrospective analysis of 6,384 patients with CHF admitted to The First Affiliated Hospital of Henan University of Science and Technology from July 1, 2017, to June 30, 2022. The definition of CHF followed the 2021 European Society of Cardiology Guidelines for the Diagnosis and Treatment of Acute and Chronic Heart Failure (2). Among the initial cohort of 6,384 patients, 1,429 were excluded in accordance with the following specified exclusion criteria: (1) age < 18 years or pregnancy; (2) severe hepatic or renal dysfunction; (3) advanced cancer or connective tissue diseases; (4) lacking data on body mass index (BMI), systolic blood pressure (SBP)/diastolic blood pressure (DBP), fasting blood glucose (FBG), triglyceride, or high-density lipoprotein cholesterol (HDL-C) at admission; and (5) in-hospital mortality or loss to follow-up. Ultimately, 4,955 patients were enrolled in this study. Furthermore, patients were categorized into four groups according to their metabolic status and BMI: metabolically healthy with normal weight (MHNW, n = 1398), metabolically unhealthy with normal weight (MUNW, n = 482), metabolically healthy with overweight or obesity (MHO, n = 1350), and metabolically unhealthy with overweight or obesity (MUO, n = 1725) (**Figure 1**).

### Ethics statement

This retrospective study was conducted in accordance with the tenets of the Declaration of Helsinki and approved by the ethics committee of The First Affiliated Hospital of Henan University of Science and Technology (2023-03-K0026). Given the retrospective design of this research, the institutional review board exempted the requirement for informed consent and ensured that all patient-related information was anonymized.

### Data collection and definitions

We gathered information on patient demographics, vital signs, medical history, laboratory test outcomes, echocardiographic data, and medication details from the electronic medical records system. Venous blood samples were collected for the analysis of laboratory indicators, including white blood cells (WBC), platelets, creatinine, serum lipid parameters, and N-terminal pro-brain natriuretic peptide (NT-proBNP), among others. The mean arterial pressure was calculated using the following formula: (SBP + 2 × DBP)/3. BMI was determined using the following formula: weight in kilograms divided by the square of height in meters, expressed as kg/m^2^. Hypertension was defined as a history of hypertension or a diagnosis at admission. Chronic kidney disease was identified by an estimated glomerular filtration rate below 60 mL/min per 1.73 m^2^, which was calculated using the Chronic Kidney Disease Epidemiology Collaboration (CKD-EPI) equation (22), or determined through medical history. Severe hepatic or renal dysfunction was defined as cirrhosis with ascites or chronic renal failure with dialysis treatment. To prevent the clinical missed diagnosis of diabetes, the diagnosis was further confirmed through the following criteria: a prior diagnosis of diabetes and/or FBG ≥7.0 mmol/L and/or random blood glucose ≥11.1 mmol/L and/or the use of hypoglycemic agents. Hypoglycemic medications included those prescribed at discharge as well as oral hypoglycemic drugs used during hospitalization, excluding SGLT2 inhibitors, as these were not exclusively used for patients with diabetes.

Metabolic status was evaluated by the presence or absence of MetS. According to the China Guidelines for Type 2 Diabetes (23) and the obesity criteria set by the Working Group on Obesity in China (24), which use BMI instead of waist circumference to assess obesity, MetS was identified by the presence of three or more of the following criteria: (1) obesity (BMI ≥ 28 kg/m^2^); (2) hyperglycemia (FBG ≥ 6.1 mmol/L and/or clinically diagnosed diabetes by physician); (3) elevated blood pressure (blood pressure ≥ 130/85 mmHg and/or clinically confirmed hypertension); (4) fasting triglyceride ≥ 1.7 mmol/L; and (5) fasting HDL-C < 1.04 mmol/L. Obesity status was categorized as normal weight (BMI < 24 kg/m^2^) or overweight/obesity (BMI ≥ 24 kg/m^2^) by the definition of the Working Group on Obesity in China (24). According to the definition of the World Health Organization (WHO), overweight or obesity was classified as having a BMI of 25 kg/m² or higher, while obesity was classified as having a BMI of 30 kg/m² or higher (24).

### Follow-up and outcomes

Prognostic data were acquired via telephone follow-ups or by examining pertinent electronic medical records over a mean follow-up duration of 3.14 ± 1.58 years. The primary outcomes of this study were all-cause mortality and cardiovascular death, with the latter primarily encompassing fatalities due to HF, sudden death, malignant arrhythmias, myocardial infarction, or other cardiac causes.

### Statistical analysis

The characteristics of the participants were delineated according to metabolic overweight/obesity phenotypes. Continuous variables are reported as the mean ± standard deviation or median with interquartile range, depending on whether the distribution was normal. For continuous data, comparisons were made using one-way analysis of variance for normally distributed data and the Kruskal−Wallis test for skewed distributions. Categorical variables are presented as frequencies and percentages, with group differences evaluated using the chi-squared or Fisher’s exact tests when appropriate.

The cumulative incidence of the primary endpoints was estimated using the Kaplan−Meier method, and differences between groups were assessed with the log-rank test. The relationship between metabolic overweight/obesity phenotypes and the incidence of primary outcomes was explored using Cox proportional hazards models. Predictors that achieved significance in univariate analyses (*P* < 0.05) (**Supplementary Table S1**) or were considered clinically important were selected as covariates in the multivariate Cox model. Furthermore, the multivariate analysis accounted for both collinearity and correlation among the variables. In addition to the unadjusted model, two other models were fitted: Model 1 controlled for age, sex, smoking status, and drinking status, and Model 2 included all variables from Model 1 with additional adjustments for New York Heart Association classification, left ventricular ejection fraction, NT-proBNP, creatinine, LDL-C, previous MI, atrial fibrillation, COPD, past CABG, ACEI/ARB/ARNI, β-blockers, diuretics, SGLT2 inhibitors, and other antidiabetic therapy. Multiple imputations with chained equations were utilized to handle missing covariates. The proportional hazards assumption was assessed through Schoenfeld residuals, revealing no observed potential violations. In this study, we conducted stratified analyses among different subgroups based on sex (male or female) and age (< 60 years or ≥ 60 years). Additionally, we performed sensitivity analysis, excluding the subset of the population potentially classified as having cardiac cachexia (BMI ≤ 20 kg/m^2^) (25), to test the consistency of the results. Finally, we reclassified and further analyzed the metabolic overweight/obesity phenotypes based on the definition of overweight and obesity set by the WHO.

All the statistical analyses were performed using R software (version 4.4.0; R Foundation for Statistical Computing, Vienna, Austria). A two-tailed *P* value <0.05 was considered to indicate significance.

## Results

### Participant characteristics

**Table 1** details the baseline characteristics of the study population categorized by metabolic overweight/obesity phenotypes. In total, 4,955 eligible participants were included in the analysis.

**Table 1:** Baseline characteristics of the study population according to metabolic overweight/obesity phenotypes.

Characteristics	MHNW	MUNW	MHO	MUO	*P* value
	(n=1398)	(n=482)	(n=1350)	(n=1725)	
Demographics					
Age (years)	66.0 (55.2-74.0)	68.0 (57.0-77.0)	66.0 (55.0-74.0)	67.0 (57.0-77.0)	**<0.001**
Male (%)	869 (62.16%)	295 (61.20%)	850 (62.96%)	1062 (61.57%)	0.849
Medical measurements					
MAP (mmHg)	93.3 (84.0-101.0)	98.7 (93.0-107.2)	94.7 (87.0-102.3)	101.0 (92.7-109.0)	**<0.001**
HR (bpm)	77.0 (66.0-86.0)	76.0 (68.0-84.0)	76.0 (66.0-85.0)	76.0 (66.0-84.0)	0.076
BMI (kg/m^2^)	21.4 (19.7-22.5)	21.5 (20.0-23.1)	26.3 (24.9-27.7)	28.4 (25.8-31.7)	**<0.001**
Current/ex-Smoker (%)	446 (31.90%)	157 (32.57%)	443 (32.81%)	573 (33.22%)	0.891
Current/ex-Drinker (%)	259 (18.53%)	82 (17.01%)	245 (18.15%)	348 (20.17%)	0.312
NYHA classification (%)					<0.001
I-II	723 (51.72%)	213 (44.19%)	630 (46.67%)	730 (42.32%)	
III	384 (27.47%)	153 (31.74%)	417 (30.89%)	572 (33.16%)	
IV	291 (20.82%)	116 (24.07%)	303 (22.44%)	423 (24.52%)	
Medical history (%)					
AF	363 (25.97%)	138 (28.63%)	344 (25.48%)	479 (27.77%)	0.343
CKD	362 (25.89%)	153 (31.74%)	356 (26.37%)	542 (31.42%)	**<0.001**
COPD	179 (12.80%)	70 (14.52%)	179 (13.26%)	287 (16.64%)	**0.010**
Diabetes	363 (25.97%)	319 (66.18%)	380 (28.15%)	1075 (62.32%)	**<0.001**
Hypertension	747 (53.43%)	370 (76.76%)	779 (57.70%)	1365 (79.13%)	**<0.001**
Previous MI	357 (25.54%)	158 (32.78%)	365 (27.04%)	568 (32.93%)	**<0.001**
Past PCI	386 (27.61%)	155 (32.16%)	409 (30.30%)	614 (35.59%)	**<0.001**
Past CABG	24 (1.72%)	8 (1.66%)	19 (1.41%)	35 (2.03%)	0.628
Laboratory measurements					
WBC (10^9^/L)	6.30 (5.11-7.90)	6.65 (5.30-8.51)	6.24 (5.03-7.88)	6.51 (5.31-8.18)	**<0.001**
Platelets (10^9^/L)	201.0 (160.0-242.0)	205.0 (163.2-246.0)	199.0 (162.0-245.0)	207.0 (167.0-254.0)	**0.007**
ALT (U/L)	24.0 (17.0-36.0)	24.0 (16.0-34.0)	24.0 (17.0-38.0)	25.0 (17.0-40.0)	0.227
AST (U/L)	23.0 (18.0-32.0)	22.0 (17.0-32.0)	24.0 (18.0-33.0)	23.0 (17.0-34.0)	0.320
Creatinine (umol/L)	70.5 (58.1-84.6)	72.5 (60.0-88.0)	70.5 (59.9-84.3)	73.0 (59.9-88.0)	**0.002**
eGFR (ml/min/1.73m^2^)	89.2 (73.5-100.5)	85.9 (68.2-100.8)	89.7 (73.4-101.2)	86.8 (69.0-99.6)	**<0.001**
FBG (mmol/L)	5.19 (4.65-6.13)	6.74 (5.62-8.65)	5.38 (4.78-6.35)	6.46 (5.34-8.02)	**<0.001**
TC (mmol/L)	3.88 (3.24-4.72)	4.06 (3.34-4.88)	3.92 (3.29-4.64)	4.05 (3.32-4.83)	**0.002**
TG (mmol/L)	1.08 (0.81-1.46)	1.77 (1.24-2.36)	1.13 (0.84-1.52)	1.82 (1.12-2.20)	**<0.001**
LDL-C (mmol/L)	2.20 (1.68-2.84)	2.34 (1.72-2.95)	2.26 (1.70-2.83)	2.37 (1.82-3.02)	**<0.001**
HDL-C (mmol/L)	1.18 (1.05-1.38)	0.91 (0.79-0.98)	1.15 (1.06-1.35)	0.87 (0.76-1.08)	**<0.001**
Potassium (mmol/L)	3.92 (3.62-4.27)	3.89 (3.61-4.28)	3.91 (3.64-4.27)	3.91 (3.65-4.28)	0.830
Sodium (mmol/L)	141.1 (138.6-143.4)	140.6 (138.7-142.9)	141.3 (138.6-143.4)	141.3 (139.0-143.5)	0.211
NT-proBNP (pg/ml)	1415.5 (733.0-4431.5)	1687.0 (704.2-5535.8)	1405.0 (794.0-3482.5)	1567.0 (824.0-4567.0)	**<0.001**
Echocardiography					
LVEF (%)	45.0 (36.0-57.0)	47.0 (36.0-59.0)	44.5 (35.0-57.0)	47.0 (36.0-58.0)	0.272
Medications (%)					
Antiplatelet agents	817 (58.44%)	324 (67.22%)	818 (60.59%)	1202 (69.68%)	**<0.001**
ACEI/ARB/ARNI	714 (51.07%)	265 (54.98%)	732 (54.22%)	982 (56.93%)	**0.013**
Beta-blocker	997 (71.32%)	381 (79.05%)	990 (73.33%)	1354 (78.49%)	**<0.001**
Statins	830 (59.37%)	336 (69.71%)	881 (65.26%)	1275 (73.91%)	**<0.001**
CCB	183 (13.09%)	92 (19.09%)	177 (13.11%)	349 (20.23%)	**<0.001**
Digoxin	194 (13.88%)	83 (17.22%)	222 (16.44%)	257 (14.90%)	0.162
Mineralocorticoid antagonists	958 (68.53%)	347 (71.99%)	929 (68.81%)	1230 (71.30%)	0.201
Diuretics	842 (60.23%)	326 (67.63%)	838 (62.07%)	1206 (69.91%)	**<0.001**
SGLT2 inhibitors	153 (10.94%)	111 (23.03%)	167 (12.37%)	360 (20.87%)	**<0.001**
Insulin	71 (5.08%)	64 (13.28%)	78 (5.78%)	205 (11.88%)	**<0.001**
Other oral antidiabetic agents	219 (15.67%)	177 (36.72%)	228 (16.89%)	608 (35.25%)	**<0.001**
Outcomes					
All-cause death	341 (24.39%)	180 (37.34%)	226 (16.74%)	641 (37.16%)	**<0.001**
CV death	199 (14.23%)	119 (24.69%)	128 (9.48%)	369 (21.39%)	**<0.001**

MHNW, metabolically healthy with normal weight; MUNW, metabolically unhealthy with normal weight; MHO, metabolically healthy with overweight or obesity; MUO, metabolically unhealthy with overweight or obesity; MAP, mean arterial pressure; HR, heart rate; BMI, body mass index; NYHA, New York Heart Association; AF, atrial fibrillation; CKD, chronic kidney disease; COPD, chronic obstructive pulmonary disease; MI, myocardial infarction; PCI, percutaneous coronary intervention; CABG, coronary artery bypass grafting; WBC, white blood cell; ALT, alanine aminotransferase; AST, aspartate aminotransferase; eGFR, estimated glomerular filtration rate; FBG, fasting blood glucose; TC, total cholesterol; TG, triglyceride; LDL-C, low-density lipoprotein cholesterol; HDL-C, high-density lipoprotein cholesterol; NT-proBNP, N-terminal pro-brain natriuretic peptide; LVEF, left ventricular ejection fraction; ACEI/ARB/ARNI, angiotensin converting enzyme inhibitor/angiotensin receptor blocker/angiotensin receptor-neprilysin inhibitors; CCB, calcium channel blockers; SGLT2, inhibitors sodium-glucose co-transporter-2 inhibitors; CV, death cardiovascular death. P values <0.05 are presented in bold.

The mean age was 65.4 years, and males accounted for 62.1%. Of these, 28.2% (n = 1398) were classified as MHNW, 9.7% (n = 482) as MUNW, 27.3% (n = 1350) as MHO, and 34.8% (n = 1725) as MUO.

Overall, the average age of the individuals in the metabolically unhealthy groups (MUNW and MUO) was greater than that of the individuals in the metabolically healthy groups (MHNW and MHO). Although there was a marginally greater proportion of females in the former groups than in the latter groups, this difference did not reach significance. The metabolic parameters, such as the mean arterial pressure, FBG, and lipid levels (excluding HDL-C), were elevated in the metabolically unhealthy groups compared to those in the metabolically healthy groups, whereas HDL-C exhibited an inverse association (all *P <*0.05). BMI levels were significantly greater in the overweight or obesity groups (MHO and MUO) compared to the normal weight groups (MHNW and MUNW). Regarding other laboratory parameters, the metabolically unhealthy groups exhibited higher WBC, platelets, and serum creatinine levels and lower eGFR (all *P <*0.05). As expected, the risk of comorbidities, including chronic kidney disease, chronic pulmonary disease, diabetes, hypertension, previous myocardial infarction, and a history of PCI, was greater in the metabolically unhealthy groups compared to the metabolically healthy groups (all *P <*0.05). Although there were no discernible differences in left ventricular ejection fraction (*P* = 0.272) between the metabolically healthy and unhealthy groups, the latter demonstrated elevated New York Heart Association classification and NT-proBNP levels in comparison to the former (all *P <*0.05). Regarding medications, a greater proportion of patients in the metabolically unhealthy groups than in the metabolically healthy groups used antiplatelet agents, ACEIs/ARBs/ARNIs, beta-blockers, statins, calcium channel blockers, diuretics, or hypoglycemic agents (all *P <*0.05).

### Association between metabolic overweight/obesity phenotypes and risk outcomes

After a mean follow-up of 3.14 years, there were 1,388 (28.0%) all-cause mortality events and 815 (16.4%) CV mortality events. The incidence rates (per 1,000 person-years) of the primary outcomes differed significantly between the metabolically healthy and unhealthy groups. Regarding all-cause mortality, the rates were 136.11 in the MUNW group and 118.54 in the MUO group, compared to 81.83 in the MHNW group and 48.38 in the MHO group. Similarly, CV mortality rates were also higher in the metabolically unhealthy groups, with rates of 89.98 in the MUNW group and 68.24 in the MUO group versus 47.75 in the MHNW group and 27.4 in the MHO group.

**Figure 2:**
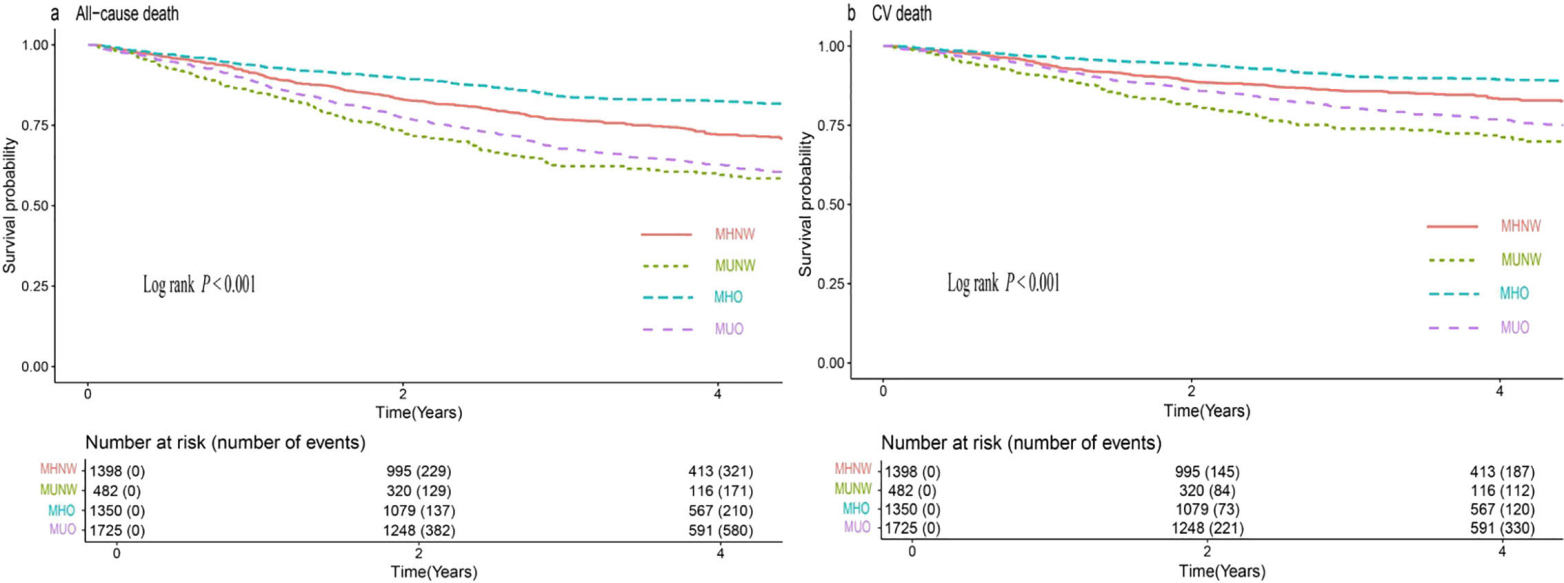
Kaplan-Meier estimation of **(A)** all-cause death and **(B)** CV death by metabolic overweight/obesity phenotypes in patients with HF. CV death, cardiovascular death; HF, heart failure; MHNW, metabolically healthy with normal weight; MUNW, metabolically unhealthy with normal weight; MHO, metabolically healthy with overweight or obesity; MUO, metabolically unhealthy with overweight or obesity.

**Figure 2** displays the Kaplan−Meier curves depicting the incidence of primary outcomes, including all-cause and CV mortality, across different metabolic overweight/obesity phenotypes. The results demonstrated that individuals identified as metabolically unhealthy (MUNW and MUO) exhibited a greater risk of primary events than did those in the MHNW group, irrespective of obesity status. Conversely, individuals in the MHO group displayed lower adverse outcome risk (log-rank test, both *P* < 0.001).

**Table 2** shows the results of the univariate and multivariate Cox proportional hazards regression analyses in the four groups. According to an unadjusted model, compared to the MHNW group, which was used as the reference group, the metabolically unhealthy groups demonstrated significantly greater hazard ratios (HRs) for all-cause mortality, regardless of obesity status (HR, 1.66 [95% CI, 1.39–1.99] for the MUNW group and HR, 1.45 [95% CI, 1.27–1.66] for the MUO group). Conversely, the protective effects of having overweight or obesity were present in the MHO group (HR, 0.60 [95% CI, 0.51–0.71]) but dissipated in the MUO group (HR, 1.45 [95% CI, 1.27–1.66]). Even after adjusting for confounding variables in two different models, the results remained unchanged: patients in the metabolically unhealthy groups still faced a significantly greater risk of all-cause death (aHR, 1.66 [95% CI, 1.38-2.00] among MUNW group and aHR, 1.42 [95% CI, 1.24-1.63] among MUO group), while those in the MHO group continued to show a reduced risk (aHR, 0.61 [95% CI, 0.51-0.72]). Consistent outcomes were observed in the multivariate Cox proportional hazards analysis assessing the impact of metabolic overweight/obesity phenotypes on CV death. The aHRs and 95% CIs were 1.91 [1.51–2.41] for the MUNW group, 1.43 [1.19–1.71] for the MUO group, and 0.59 [0.47–0.73] for the MHO group.

**Table 2:** HRs (95% CI) of primary outcomes according to metabolic overweight/obesity phenotypes.

Categories	Incidence/ 1000 person-y	Unadjusted		Model 1		Model 2	
		HR (95% CI)	*P*-value	HR (95% CI)	*P*-value	HR (95% CI)	*P*-value
All-cause death							
MHNW (n=1398)	81.83	Ref.		Ref.		Ref.	
MUNW (n=482)	136.11	1.66 (1.39-1.99)	**<0.001**	1.64 (1.37-1.97)	**<0.001**	1.66 (1.38-2.00)	**<0.001**
MHO (n=1350)	48.38	0.60 (0.51-0.71)	**<0.001**	0.60 (0.51-0.72)	**<0.001**	0.61 (0.51-0.72)	**<0.001**
MUO (n=1725)	118.54	1.45 (1.27-1.66)	**<0.001**	1.43 (1.25-1.63)	**<0.001**	1.42 (1.24-1.63)	**<0.001**
CV death							
MHNW (n=1398)	47.75	Ref.		Ref.		Ref.	
MUNW (n=482)	89.98	1.88 (1.50-2.36)	**<0.001**	1.86 (1.48-2.33)	**<0.001**	1.91 (1.51-2.41)	**<0.001**
MHO (n=1350)	27.4	0.59 (0.47-0.73)	**<0.001**	0.58 (0.47-0.74)	**<0.001**	0.59 (0.47-0.73)	**<0.001**
MUO (n=1725)	68.24	1.44 (1.21-1.71)	**<0.001**	1.42 (1.20-1.70)	**<0.001**	1.43 (1.19-1.71)	**<0.001**

HR, hazard ratio; CI, confidence interval; MHNW, metabolically healthy with normal weight; MUNW, metabolically unhealthy with normal weight; MHO, metabolically healthy with overweight or obesity; MUO, metabolically unhealthy with overweight or obesity; CV death, cardiovascular death. P values <0.05 are presented in bold.

Model 1: adjusted for age, sex, smoking status, drinking status.

Model 2: adjusted for Model 1 + NYHA classification, LVEF, NT-proBNP, creatinine, LDL-C, previous MI, atrial fibrillation, COPD, past CABG, ACEI/ARB/ARNI, β-blocker, diuretics, SGLT2 inhibitors and other antidiabetic therapy.

### Association of metabolic overweight/obesity phenotypes with mortality across age- and sex-stratified subgroups

We further conducted exploratory analyses in subgroups stratified by age and sex. Among both males and elderly females (aged ≥ 60 years), Kaplan–Meier analysis indicated that compared with individuals in the MHNW group, those in the MUNW and MUO groups had a greater risk of all-cause mortality, regardless of obesity status. However, individuals classified as MHO demonstrated a lower risk of all-cause mortality. In the subgroup of nonelderly females (aged < 60 years), we observed a different phenomenon: the detrimental effects of MetS were markedly diminished in the MUNW group or disappeared in the MUO group, while the protective effects of having overweight or obesity remained consistent across both the MHO and MUO groups, irrespective of metabolic status. The aforementioned findings remained consistent when CV death was used as the study endpoint (**Supplementary Figure S1**).

**Figure 3:**
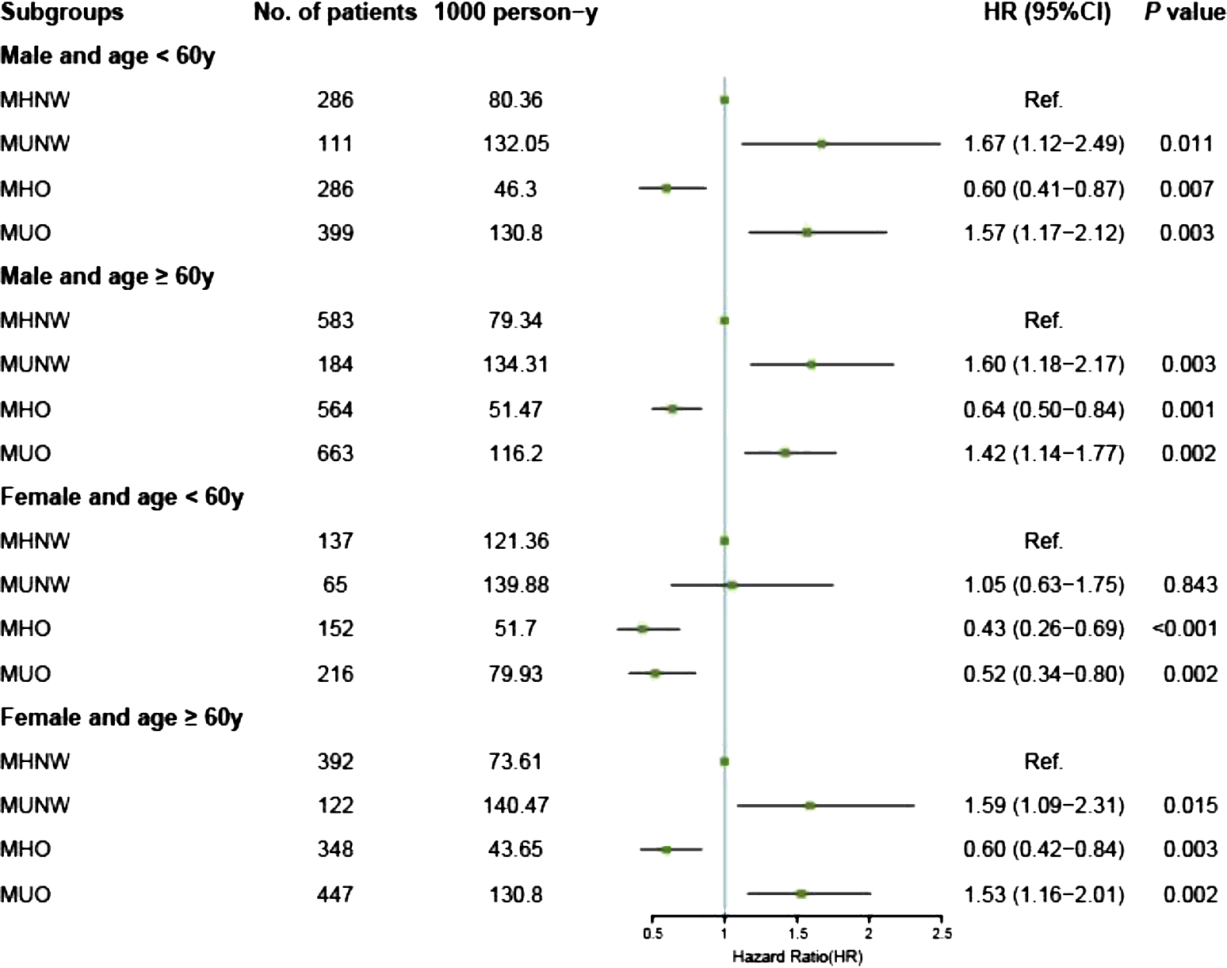
Forest plot of all-cause death according to metabolic overweight/obesity phenotypes in patients with HF adjusted for model 2. HR, hazard ratio; CI, confidence interval; HF, heart failure; MHNW, metabolically healthy with normal weight; MUNW, metabolically unhealthy with normal weight; MHO, metabolically healthy with overweight or obesity; MUO, metabolically unhealthy with overweight or obesity.

**Figure 4:**
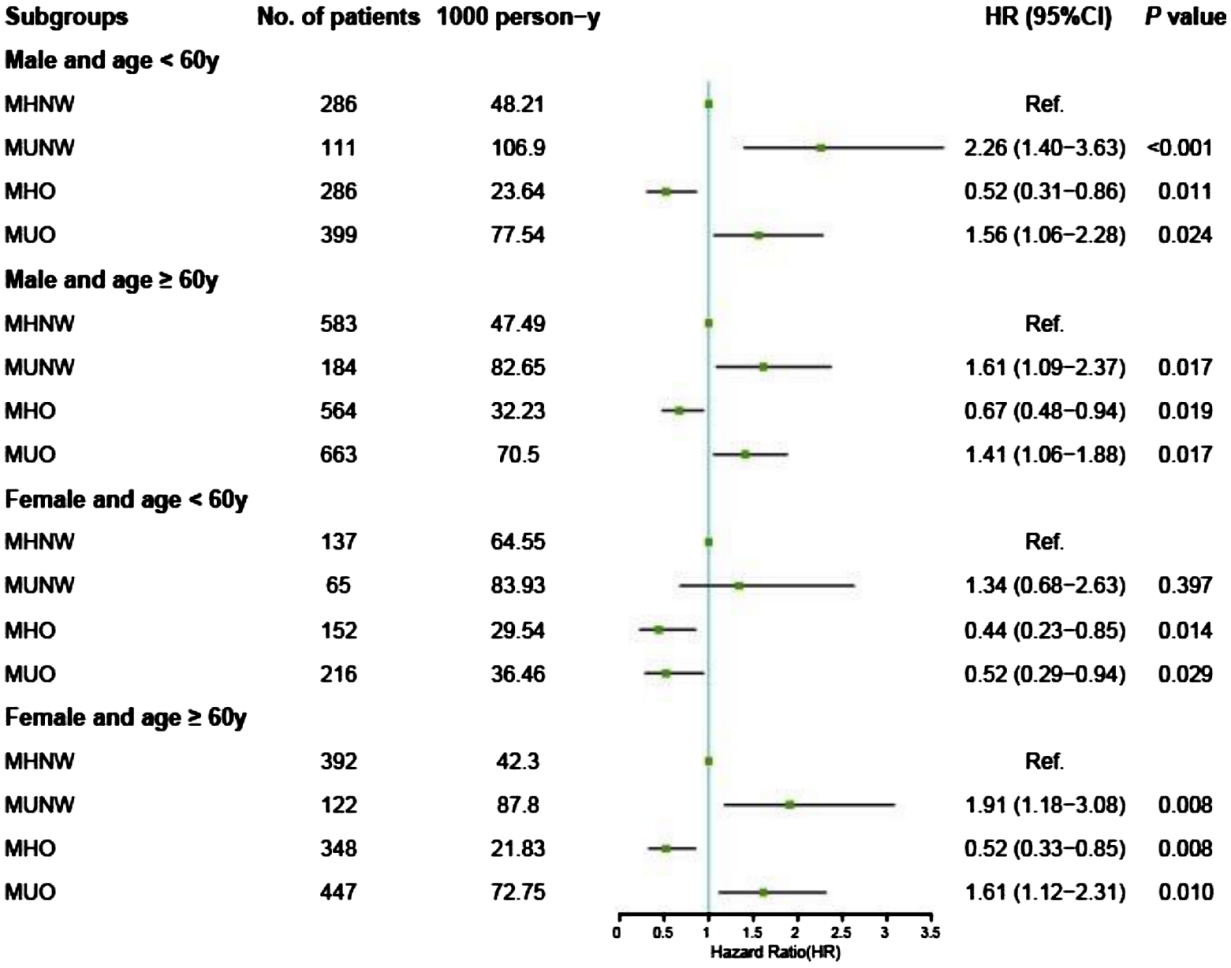
Forest plot of CV death according to metabolic overweight/obesity phenotypes in patients with HF adjusted for model 2. CV death, cardiovascular death; HR, hazard ratio; CI, confidence interval; HF, heart failure; MHNW, metabolically healthy with normal weight; MUNW, metabolically unhealthy with normal weight; MHO, metabolically healthy with overweight or obesity; MUO, metabolically unhealthy with overweight or obesity.

The results of the Cox proportional hazards analyses of the associations between metabolic overweight/obesity phenotypes and primary outcomes among different subgroups according to age and sex are presented in **Supplementary Table S2**. Consistent with the Kaplan–Meier analysis results, among males and elderly females, adverse prognostic risks persisted in the metabolically unhealthy groups (MUNW and MUO) compared to those in the MHNW group, irrespective of obesity status, even after multivariable adjustment (all *P* < 0.05). In contrast, individuals classified as MHO demonstrated a more favorable prognosis with MHNW as a reference in the aforementioned groups: for males under 60 years of age, the aHRs were 0.60 [0.41–0.87] for all-cause mortality and 0.52 [0.31–0.86] for CV mortality; for males aged 60 years and older, the aHRs were 0.64 [0.50–0.84] for all-cause mortality and 0.67 [0.48–0.94] for CV mortality; similarly, for females aged 60 years and older, the aHRs were 0.60 [0.42–0.84] for all-cause mortality and 0.52 [0.33–0.85] for CV mortality (**Figures 3**, **4**).

However, different phenomena were observed among nonelderly females. First, the adverse prognostic effects of MetS were absent across all obesity statuses: the MUNW group exhibited aHRs of 1.05 [0.63–1.75] for all-cause mortality and 1.34 [0.68–2.63] for CV mortality (all *P* > 0.05); the MUO group displayed aHRs of 0.52 [0.34–0.80] for all-cause mortality and 0.52 [0.29–0.94] for CV mortality. Second, the protective effects of having overweight or obesity persisted and remained unaffected by MetS: the MHO group had an aHR of 0.43 [0.26–0.69] for all-cause mortality and 0.44 [0.23–0.85] for CV mortality; similarly, the MUO group had an aHR of 0.52 [0.34–0.80] for all-cause mortality and 0.52 [0.29–0.94] for CV mortality (**Figures 3**, **4**, **Supplementary Table S2**). Additionally, we conducted a sensitivity analysis by excluding the population potentially characterized as having cardiac cachexia, identified by a BMI of ≤ 20 kg/m^2^ (25). The results demonstrated that the conclusions remained unchanged both in the overall cohort and across all subgroups (**Supplementary Table S3**).

### Association between metabolic overweight/obesity phenotypes based on the WHO definition and primary outcomes

We reclassified and conducted further analyses on the study population based on the WHO’s definition of overweight and obesity. No substantial changes were observed within the overall population: for the MUNW group, the aHRs were 1.70 [1.45–1.98] for all-cause mortality and 1.87 [1.54–2.28] for CV mortality; for the MHO group, the aHRs were 0.61 [0.51–0.73] for all-cause mortality and 0.52 [0.41–0.66] for CV mortality; for the MUO group, the aHRs were 1.49 [1.30–1.70] for all-cause mortality and 1.46 [1.22–1.74] for CV mortality, with the MHNW group as a reference. In further analyses stratified by age and sex, the association of metabolic overweight/obesity phenotypes with either all-cause mortality or CV death remained consistent (**Supplementary Table S4**).

## Discussion

In this study, we examined the association between metabolic overweight/obesity phenotypes and mortality among patients with CHF, with an additional focus on various subgroups delineated by age and sex. The findings suggest that MetS is closely associated with poor prognosis in patients with CHF independent of overweight or obesity status. In contrast, the influence of the obesity paradox was markedly affected by MetS, with the paradox only occurring in patients without MetS. These findings were primarily observed in males and elderly females. Interestingly, further observations revealed that in nonelderly females, the adverse effects of MetS were significantly diminished or entirely absent, whereas the protective effects of having overweight or obesity continued irrespective of metabolic status.

MetS represents a constellation of metabolic disorders with a high prevalence in the general population and is intricately linked with the development and progression of HF (5). Multiple studies have demonstrated that MetS is closely associated with the incidence of HF. Due to regional disparities, population characteristics, and variations in definitions, the prevalence of MetS among patients with HF ranged from 37% to 78.8%, indicating a generally high incidence trend (26). A Japanese cohort study of 3,603 patients showed that the incidence of MetS among patients with CHF is over twice that of the general population (6). The probability that MetS leads to HF may be linked to its core components and fundamental changes, particularly IR. First, long-term hypertension can result in HF through various mechanisms, including concentric hypertrophy, myocardial insult, eccentric hypertrophy, and imbalances in the neurohumoral regulation system of the body (27). Second, hyperlipidemia may induce HF by promoting oxidative stress and inflammatory cardiac fibrosis, reducing autophagy and microvascular density in cardiac myocytes, altering mitochondrial function in myocardial cells, and ultimately leading to cardiac dysfunction and electrophysiological alterations (28). Third, diabetes-induced hyperglycemia and hyperinsulinemia can cause capillary damage, myocardial fibrosis, and myocardial hypertrophy with mitochondrial dysfunction (29). Fourth, IR may lead to HF through several pathways, including mitochondrial dysfunction in myocardial cells, reduced cardiac efficiency, increased oxidative stress, inflammation, elevated apoptosis, and myocardial fibrosis (8). Similarly, HF can impair insulin sensitivity through mechanisms such as the excessive stimulation of β-adrenergic receptors, thereby triggering or exacerbating IR and creating a vicious cycle (30).

Although MetS is closely and significantly associated with the incidence and progression of HF, studies on the association between MetS and the prognosis of patients with HF have yielded inconsistent results. A cohort study of an Asian population included 4,762 patients with CHF, 41.3% of whom had MetS. Over a follow-up period of 3.2 ± 1.1 years, the study revealed that MetS was associated with an increased incidence of composite endpoints of all-cause mortality and atherosclerotic events in male patients in this cohort (aHR, 1.28 [95% CI, 1.06–1.54], *P* = 0.011) (10). Another study involving 865 indigent patients with HF revealed that during an average follow-up period of 2.6 ± 2.2 years, the mortality rate among those with MetS was 24%, compared to 16% among those without MetS. After multivariate adjustment, the relative risk of death associated with MetS was 1.5 (95% CI: 1.1–2.1) (11). However, other studies revealed different results. One study of HF in a Korean population indicated that although individuals with MetS face increased cardiovascular risk, their mortality rate from HF is relatively lower (13). A meta-analysis encompassing 10 studies with a total of 18,590 patients with HF indicated that MetS was not associated with all-cause mortality (HR, 1.04 [95% CI, 0.88–1.23]) but increased the risk of composite cardiovascular events (HR, 1.73 [95% CI, 1.23–2.45]) (31). The variations across study results may stem from differences in the definition of metabolic disturbances and the composition of study populations and their metabolic characteristics. Therefore, further research remains necessary to reach a consensus on these controversial phenomena.

Patients who have overweight or obesity exhibit increased susceptibility to various metabolic impairments (32) and are more likely to exhibit predispositions toward MetS. Additionally, numerous studies have confirmed that having overweight or obesity is a risk factor for the development of HF. A Mendelian randomization analysis incorporating two principal Danish cohorts and additional genetic data from extensive databases such as GIANT, HERMES, and the UK Biobank established a significant causal link between BMI and HF (18). The analysis revealed that every 1 kg/m^2^ increase in BMI was associated with a 39% greater risk of HF, with a causal risk ratio of 1.39 [95% CI: 1.27–1.52]. Another study included 4,033 individuals with obesity who had no history of HF at baseline; 2,003 underwent bariatric surgery, and the remaining 2,030 received usual care. Over a median follow-up period of 22 years, the incidence of HF was significantly lower in the surgical group than in the usual care group (HR, 0.65 [95% CI, 0.54–0.79], *P* < 0.001) (33). This finding suggests that the risk of HF decreases with the extent of weight loss. The mechanisms by which individuals with elevated BMI are susceptible to HF are diverse and can be classified into indirect and direct pathways (34). Indirect pathways involve a heightened risk of conditions such as hypertension, diabetes, and hyperlipidemia associated with overweight and obesity (35). These conditions are intimately connected to cardiovascular diseases and significantly increase the likelihood of HF. Direct mechanisms involve tissue inflammation, endothelial dysfunction, alterations in hemodynamics, and increased sympathetic nerve activity, which collectively lead to myocardial remodeling and the subsequent onset of HF (34).

Some studies have suggested the existence of an obesity paradox regarding the correlation between overweight or obesity and the prognosis of patients with HF. This obesity paradox posits that although elevated body weight is associated with an increased incidence of HF, it correlates with more favorable prognostic outcomes (5). Several possible mechanisms for the obesity paradox phenomenon have been proposed (36): it is suggested that patients with obesity may maintain higher levels of glucose and metabolic substrates, reduced sympathetic activity, and lower norepinephrine levels, which could compensate for the adverse effects of high metabolism and high energy expenditure caused by HF. However, some researchers have challenged the idea of this paradoxical phenomenon, suggesting that a higher BMI may be linked to increased mortality rates in patients with HF or raising doubts about the universality of the obesity paradox, proposing that it might only apply to certain specific subgroups (17–19).

Currently, the associations between metabolic dysregulation and overweight or obesity and the prognosis of patients with HF remain controversial and uncertain. Although MetS and overweight or obesity can influence each other and often coexist, this is not always the case. Many patients exhibit either isolated metabolic disorders or obesity, resulting in different metabolic overweight/obesity phenotypes. Previous studies on metabolically unhealthy phenotypes and their prognoses have primarily focused on populations with cancer or nonheart failure conditions (37–39), with few studies investigating the relationship between these phenotypes and the prognosis of patients with HF. Our research revealed that in the overall population with CHF, the adverse effects of MetS on prognosis do not change with alterations in obesity status, indicating that the MUNW and MUO phenotypes are closely associated with mortality risk. We observed that the obesity paradox is only present in the MHO phenotype. The primary reason behind this phenomenon is the higher metabolic compensation capacity in individuals with obesity, with skeletal muscle playing a crucial role. In patients with chronic diseases, skeletal muscle atrophy is significantly influenced by metabolic disorders (40), whereas in metabolically healthy patients with HF, the increased weight proportion is likely attributed to non-fat tissues such as skeletal muscle. As a major reservoir for glucose and protein, skeletal muscle plays an important role in energy and metabolic supplementation (41) and is closely associated with the prognosis of patients with HF (42). However, the obesity paradox is clearly influenced by metabolic status, disappearing within MetS presence. We propose that the following two factors might explain this phenomenon. First, patients with MetS exhibit elevated IR and may be in a state of chronic inflammation (43). Their energy metabolism expenditure could be more pronounced than that of patients without MetS, potentially offsetting the energy storage benefits associated with overweight or obesity. Second, in the context of the obesity paradox, energy storage and the amelioration of chronic disease outcomes may be primarily facilitated by an increase in muscle mass or subcutaneous fat rather than visceral adipose tissue (VAT) (44, 45). However, MetS is closely associated with increased VAT (46). Therefore, in individuals with overweight or obesity and MetS, the increase may predominantly be in VAT content rather than muscle mass or subcutaneous fat. VAT can adversely affect cardiovascular diseases (47), leading to the disappearance of the obesity paradox in individuals with MetS.

Finally, given that metabolic levels may vary across different age and sex groups (20, 21), we conducted a stratified exploratory analysis by age and sex. The results of the exploratory analysis indicate that among males and elderly females, the following conclusions still hold: the adverse prognostic impacts of MetS are unaffected by obesity status, while the obesity paradox is significantly influenced by metabolic status. Interestingly, in nonelderly females, we observed a completely different phenomenon: the detrimental effects of MetS disappeared regardless of obesity status, and the obesity paradox persisted without being influenced by metabolic status. We speculated that the primary reason for this phenomenon may be associated with the relatively higher levels of estrogen in nonelderly females. First, the protective effects of estrogen on the cardiovascular system (48) could partially counterbalance the detrimental impacts of adverse metabolic conditions. Second, estrogen can influence fat distribution, notably by favoring peripheral rather than visceral fat accumulation (49), which may lessen or nullify the negative effects of MetS on visceral fat distribution. Third, although we hypothesize that estrogen levels may reduce the adverse effects of MetS and intensify the manifestations of the obesity paradox, we currently do not recommend estrogen supplementation for such patients due to the lack of support from large-scale clinical trial data and the potential risks of increased incidence of diseases such as breast and ovarian cancers (50). Finally, we reclassified and analyzed the study population based on the WHO definition, observing no significant changes in the outcomes. This supported the applicability of our findings across various ethnic groups.

### Limitations

This study has several limitations. First, despite the large sample size, the investigation did not comprehensively track the evolution of metabolic status in the enrolled patients owing to insufficient information during the follow-up period. Second, the follow-up phase might be subject to some degree of recall or reporting bias, especially regarding the date and cause of death. Third, inherent to observational studies, unmeasured confounding factors could influence the outcomes, necessitating cautious interpretation of the results. Fourth, this study was fundamentally observational; hence, it was impossible to establish a causal relationship between the exposure factors and the observed outcomes. Fifth, we were unable to obtain measurements of skeletal muscle mass, thus precluding an evaluation of its prognostic value in patients with obesity. Finally, as this was a retrospective study relying on existing electronic medical records, the accuracy of baseline information was potentially limited. This could have affected the interpretation of results, and such limitations should have been considered when analyzing the findings. Future studies could consider employing a prospective design to enhance data quality.

## Conclusions

In patients with CHF, the prognostic effects of MetS and overweight or obesity interact and are influenced by age and sex. In males and elderly females, the detrimental impacts of MetS surpass the protective advantages offered by overweight or obesity. Conversely, in nonelderly females, the protective effects of having overweight or obesity significantly outweigh the negative consequences of metabolic disorder. These findings emphasize the importance of prioritizing the management of metabolic disorders within specific populations. Additionally, in order to effectively improve patient outcomes, further research is necessary to understand the underlying mechanisms of the difference in the relationships between overweight or obesity and survival among patients with heart failure concomitant with MetS.

## Data Availability

The datasets used and analyzed during the current study are available from the corresponding author upon reasonable request. Requests to access the datasets should be directed to KH, kunlunhe_301@163.com.

## Supplementary material

Supplementary Figure 1 Kaplan–Meier estimation of all-cause death and CV death by metabolic overweight/obesity phenotypes among different subgroups: **(A1)** all-cause death in male and age < 60y group, **(A2)** CV death in male and age < 60y group, **(B1)** all-cause death in male and age ≥ 60y group, **(B2)** CV death in male and age ≥ 60y group, **(C1)** all-cause death in female and age < 60y group, **(C2)** CV death in female and age < 60y group, **(D1)** all-cause death in female and age ≥ 60y group, **(D2)** CV death in female and age ≥ 60y group. *CV death* cardiovascular death, *MHNW* metabolically healthy with normal weight, *MUNW* metabolically unhealthy with normal weight, *MHO* metabolically healthy with overweight or obesity, *MUO* metabolically unhealthy with overweight or obesity.
