# Supplementary figures and images for: Associations between metabolic overweight/obesity phenotypes and mortality risk among patients with chronic heart failure
Source: Front Endocrinol (Lausanne). 2024 Sep 20;15:1445395. doi: 10.3389/fendo.2024.1445395 (PMC11452845; doi:10.3389/fendo.2024.1445395)

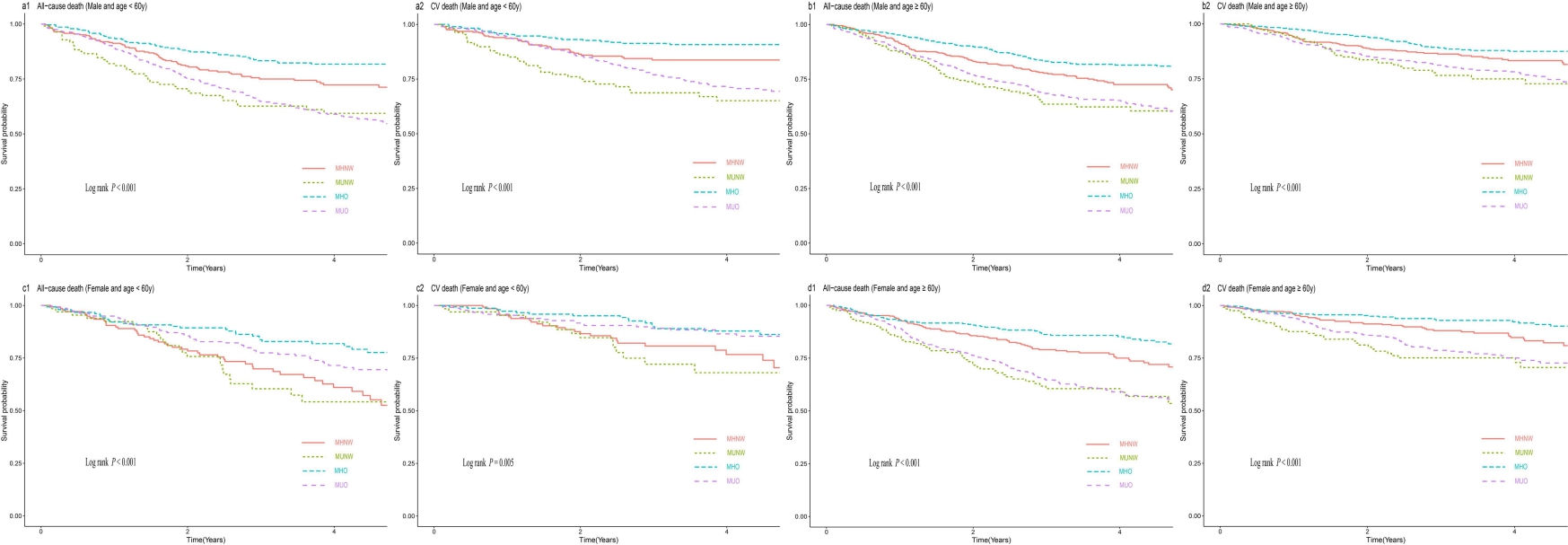

Supplement: Supplementary Figure 1 — Kaplan–Meier estimation of all-cause death and CV death by metabolic overweight/obesity phenotypes among different subgroups: (A1) all-cause death in male and age < 60y group, (A2) CV death in male and age < 60y group, (B1) all-cause death in male and age ≥ 60y group, (B2) CV death in male and age ≥ 60y group, (C1) all-cause death in female and age < 60y group, (C2) CV death in female and age < 60y group, (D1) all-cause death in female and age ≥ 60y group, (D2) CV death in female and age ≥ 60y group. CV death cardiovascular death, MHNW metabolically healthy with normal weight, MUNW metabolically unhealthy with normal weight, MHO metabolically healthy with overweight or obesity, MUO metabolically unhealthy with overweight or obesity. [file Image1.jpeg]
