# Supplementary material for: Associations between metabolic overweight/obesity phenotypes and mortality risk among patients with chronic heart failure
Source: Front Endocrinol (Lausanne). 2024 Sep 20;15:1445395. doi: 10.3389/fendo.2024.1445395 (PMC11452845; doi:10.3389/fendo.2024.1445395)
Supplement: Supplementary file 2 [file Table1.docx]

| **Table S1** Univariable analysis of the relationship between each predictor and mortality | | |
| --- | --- | --- |
| **Characteristics** | **Unadjusted HR (95% CI)** | ***P* value** |
| Clinical variables |  |  |
| Age, per SD | 1.13 (1.07-1.20) | **<0.001** |
| Male | 1.01 (0.91-1.13) | 0.855 |
| HR, per SD | 1.02 (0.96-1.07) | 0.535 |
| BMI, per SD | 1.01 (0.96-1.07) | 0.676 |
| Current/ex-Smoker | 1.05 (0.94-1.18) | 0.358 |
| Current/ex-Drinker | 1.05 (0.92-1.20) | 0.480 |
| NYHA functional class III | 1.16 (1.03-1.32) | **0.019** |
| NYHA functional class IV | 1.39 (1.21-1.59) | **<0.001** |
| Atrial fibrillation | 1.16 (1.03-1.30) | **0.013** |
| Chronic kidney disease | 1.18 (1.05-1.32) | **0.004** |
| COPD | 1.17 (1.01-1.35) | **0.034** |
| Diabetes | 1.17 (1.05-1.30) | **0.004** |
| Hypertension | 1.12 (1.01-1.26) | **0.036** |
| Previous MI | 1.15 (1.03-1.28) | **0.016** |
| Past PCI | 0.95 (0.84-1.06) | 0.329 |
| Past CABG | 1.45 (1.03-2.04) | **0.033** |
| LVEF, per SD | 0.90 (0.86-0.95) | **<0.001** |
| MUNW | 1.66 (1.39-1.99) | **<0.001** |
| MHO | 0.60 (0.51-0.71) | **<0.001** |
| MUO | 1.45 (1.27-1.66) | **<0.001** |
| Laboratory parameters |  |  |
| White blood cell, per SD | 1.03 (0.98-1.09) | 0.234 |
| Platelets, per SD | 1.01 (0.96-1.07) | 0.719 |
| ALT, per SD | 1.00 (0.95-1.05) | 0.944 |
| AST, per SD | 0.99 (0.94-1.05) | 0.882 |
| Creatinine, per SD | 1.06 (1.02-1.11) | **0.003** |
| eGFR, per SD | 0.81 (0.77-0.85) | **<0.001** |
| FBG, per SD | 1.07 (1.02-1.12) | **0.008** |
| Total cholesterol, per SD | 1.04 (0.98-1.10) | 0.137 |
| Triglyceride, per SD | 1.05 (1.00-1.11) | **0.036** |
| LDL-C, per SD | 1.04 (0.99-1.09) | 0.146 |
| HDL-C, per SD | 0.95 (0.89-1.00) | 0.052 |
| Potassium, per SD | 0.97 (0.92-1.03) | 0.326 |
| Sodium, per SD | 1.02 (0.97-1.08) | 0.423 |
| NT-proBNP, per SD | 1.12 (1.07-1.17) | **<0.001** |
| Medications |  |  |
| Antiplatelet agents | 1.06 (0.95-1.18) | 0.318 |
| ACEI/ARB/ARNI | 1.01 (0.91-1.12) | 0.836 |
| Beta-blocker | 0.85 (0.75-0.96) | **0.008** |
| Statins | 1.06 (0.94-1.18) | 0.351 |
| CCB | 1.14 (0.99-1.30) | 0.059 |
| **Table S1** (continued) |  |  |
| **Characteristics** | **Unadjusted HR (95% CI)** | ***P* value** |
| Digoxin | 1.06 (0.92-1.23) | 0.428 |
| Mineralocorticoid antagonists | 1.08 (0.96-1.22) | 0.190 |
| Diuretics | 1.15 (1.03-1.29) | **0.013** |
| SGLT2 inhibitors | 1.10 (0.95-1.27) | 0.207 |
| Insulin | 1.26 (1.05-1.50) | **0.011** |
| Other oral antidiabetic agents | 1.06 (0.94-1.19) | 0.354 |

*HR* hazard ratio, *CI* confidence interval, *SD* standard deviation, *BMI* body mass index, *NYHA* New York Heart Association, *COPD* chronic obstructive pulmonary disease, *MI* myocardial infarction, *PCI* percutaneous coronary intervention, *CABG* coronary artery bypass grafting, *LVEF* left ventricular ejection fraction, *MUNW* metabolically unhealthy with normal weight, *MHO* metabolically healthy with overweight or obesity, *MUO* metabolically unhealthy with overweight or obesity, *ALT* alanine aminotransferase, *AST* aspartate aminotransferase, *eGFR* estimated glomerular filtration rate, *FBG* fasting blood glucose, *LDL-C* low-density lipoprotein cholesterol, *HDL-C* high-density lipoprotein cholesterol, *NT-proBNP* N-terminal pro-brain natriuretic peptide, *ACEI/ARB/ARNI* angiotensin converting enzyme inhibitor/angiotensin receptor blocker/angiotensin receptor-neprilysin inhibitors, *CCB* calcium channel blockers, *SGLT2* inhibitors sodium-glucose co-transporter-2 inhibitors. *P* values < 0.05 are presented in bold.

**Table S2** HRs (95% CI) of primary outcomes in different subgroups based on age and sex

| **Subgroups** | **Incidence/**  **1000 person-y** |  | **Model 1** |  |  | **Model 2** |  |
| --- | --- | --- | --- | --- | --- | --- | --- |
|  |  |  | **HR (95% CI)** | ***P*-value** |  | **HR (95% CI)** | ***P*-value** |
| Male and age < 60 years | |  |  |  |  |  |  |
| All-cause death |  |  |  |  |  |  |  |
| MHNW (n=286) | 80.36 |  | Ref. |  |  | Ref. |  |
| MUNW (n=111) | 132.05 |  | 1.60 (1.09-2.36) | **0.017** |  | 1.67 (1.12-2.49) | **0.011** |
| MHO (n=286) | 46.3 |  | 0.59 (0.41-0.87) | **0.007** |  | 0.60 (0.41-0.87) | **0.007** |
| MUO (n=399) | 130.8 |  | 1.55 (1.16-2.06) | **0.003** |  | 1.57 (1.17-2.12) | **0.003** |
| CV death |  |  |  |  |  |  |  |
| MHNW (n=286) | 48.21 |  | Ref. |  |  | Ref. |  |
| MUNW (n=111) | 106.9 |  | 2.17 (1.38-3.43) | **<0.001** |  | 2.26 (1.40-3.63) | **<0.001** |
| MHO (n=286) | 23.64 |  | 0.51 (0.31-0.85) | **0.010** |  | 0.52 (0.31-0.86) | **0.011** |
| MUO (n=399) | 77.54 |  | 1.55 (1.08-2.25) | **0.019** |  | 1.56 (1.06-2.28) | **0.024** |
| Male and age ≥ 60 years | |  |  |  |  |  |  |
| All-cause death |  |  |  |  |  |  |  |
| MHNW (n=583) | 79.34 |  | Ref. |  |  | Ref. |  |
| MUNW (n=184) | 134.31 |  | 1.58 (1.18-2.13) | **0.002** |  | 1.60 (1.18-2.17) | **0.003** |
| MHO (n=564) | 51.47 |  | 0.65 (0.50-0.84) | **0.001** |  | 0.64 (0.50-0.84) | **0.001** |
| MUO (n=663) | 116.2 |  | 1.39 (1.12-1.72) | **0.002** |  | 1.42 (1.14-1.77) | **0.002** |
| CV death |  |  |  |  |  |  |  |
| MHNW (n=583) | 47.49 |  | Ref. |  |  | Ref. |  |
| MUNW (n=184) | 82.65 |  | 1.62 (1.11-2.37) | **0.013** |  | 1.61 (1.09-2.37) | **0.017** |
| MHO (n=564) | 32.23 |  | 0.68 (0.49-0.94) | **0.020** |  | 0.67 (0.48-0.94) | **0.019** |
| MUO (n=663) | 70.5 |  | 1.40 (1.06-1.84) | **0.016** |  | 1.41 (1.06-1.88) | **0.017** |
| Female and age < 60 years | |  |  |  |  |  |  |
| All-cause death |  |  |  |  |  |  |  |
| MHNW (n=137) | 121.36 |  | Ref. |  |  | Ref. |  |
| MUNW (n=65) | 139.88 |  | 1.12 (0.69-1.83) | 0.645 |  | 1.05 (0.63-1.75) | 0.843 |
| MHO (n=152) | 51.7 |  | 0.44 (0.27-0.70) | **<0.001** |  | 0.43 (0.26-0.69) | **<0.001** |
| MUO (n=216) | 79.93 |  | 0.60 (0.40-0.89) | **0.011** |  | 0.52 (0.34-0.80) | **0.002** |
| CV death |  |  |  |  |  |  |  |
| MHNW (n=137) | 64.55 |  | Ref. |  |  | Ref. |  |
| MUNW (n=65) | 83.93 |  | 1.26 (0.66-2.39) | 0.487 |  | 1.34 (0.68-2.63) | 0.397 |
| MHO (n=152) | 29.54 |  | 0.47 (0.25-0.88) | **0.019** |  | 0.44 (0.23-0.85) | **0.014** |
| MUO (n=216) | 36.46 |  | 0.54 (0.31-0.94) | **0.030** |  | 0.52 (0.29-0.94) | **0.029** |
| Female and age ≥ 60 years |  |  |  |  |  |  |  |
| All-cause death |  |  |  |  |  |  |  |
| MHNW (n=392) | 73.61 |  | Ref. |  |  | Ref. |  |
| MUNW (n=122) | 140.47 |  | 1.69 (1.18-2.42) | **0.004** |  | 1.59 (1.09-2.31) | **0.015** |
| MHO (n=348) | 43.65 |  | 0.59 (0.42-0.84) | **0.003** |  | 0.60 (0.42-0.84) | **0.003** |
| MUO (n=447) | 130.8 |  | 1.61 (1.24-2.10) | **<0.001** |  | 1.53 (1.16-2.01) | **0.002** |
| CV death |  |  |  |  |  |  |  |
| MHNW (n=392) | 42.3 |  | Ref. |  |  | Ref. |  |
| **Table S2** (continued) | |  |  |  |  |  |  |
| **Subgroups** | **Incidence/**  **1000 person-y** |  | **Model 1** |  |  | **Model 2** |  |
|  |  |  | **HR (95% CI)** | ***P*-value** |  | **HR (95% CI)** | ***P*-value** |
| MUNW (n=122) | 87.8 |  | 1.93 (1.22-3.05) | **0.005** |  | 1.91 (1.18-3.08) | **0.008** |
| MHO (n=348) | 21.83 |  | 0.52 (0.33-0.84) | **0.008** |  | 0.52 (0.33-0.85) | **0.008** |
| MUO (n=447) | 72.75 |  | 1.62 (1.15-2.29) | **0.006** |  | 1.61 (1.12-2.31) | **0.010** |

*HR* hazard ratio, *CI* confidence interval, *MHNW* metabolically healthy with normal weight, *MUNW* metabolically unhealthy with normal weight, *MHO* metabolically healthy with overweight or obesity, *MUO* metabolically unhealthy with overweight or obesity, *CV death* cardiovascular death. *P* values < 0.05 are presented in bold.

Model 1: adjusted for age, sex, smoking status, drinking status.

Model 2: adjusted for Model 1 + NYHA classification, LVEF, NT-proBNP, creatinine, LDL-C, previous MI, atrial fibrillation, COPD, past CABG, ACEI/ARB/ARNI, β-blocker, diuretics, SGLT2 inhibitors and other antidiabetic therapy.

**Table S3** HRs (95% CI) of primary outcomes excluding cardiac cachexia in sensitivity analysis

| **Subgroups** | **Incidence/**  **1000 person-y** |  | **Model 1** |  |  | **Model 2** |  |
| --- | --- | --- | --- | --- | --- | --- | --- |
|  |  |  | **HR (95% CI)** | ***P*-value** |  | **HR (95% CI)** | ***P*-value** |
| Overall (n=4438) |  |  |  |  |  |  |  |
| All-cause death |  |  |  |  |  |  |  |
| MHNW (n=1002) | 83.49 |  | Ref. |  |  | Ref. |  |
| MUNW (n=361) | 142.56 |  | 1.67 (1.35-2.05) | **<0.001** |  | 1.68 (1.36-2.08) | **<0.001** |
| MHO (n=1350) | 48.38 |  | 0.59 (0.50-0.71) | **<0.001** |  | 0.59 (0.49-0.70) | **<0.001** |
| MUO (n=1725) | 118.54 |  | 1.40 (1.21-1.63) | **<0.001** |  | 1.39 (1.19-1.61) | **<0.001** |
| CV death |  |  |  |  |  |  |  |
| MHNW (n=1002) | 48.28 |  | Ref. |  |  | Ref. |  |
| MUNW (n=361) | 95.04 |  | 1.92 (1.48-2.50) | **<0.001** |  | 2.00 (1.53-2.61) | **<0.001** |
| MHO (n=1350) | 27.4 |  | 0.58 (0.46-0.74) | **<0.001** |  | 0.58 (0.46-0.74) | **<0.001** |
| MUO (n=1725) | 68.24 |  | 1.41 (1.17-1.72) | **<0.001** |  | 1.42 (1.16-1.73) | **<0.001** |
| Male and age < 60 years | |  |  |  |  |  |  |
| All-cause death |  |  |  |  |  |  |  |
| MHNW (n=208) | 82.08 |  | Ref. |  |  | Ref. |  |
| MUNW (n=75) | 146.08 |  | 1.74 (1.11-2.74) | **0.016** |  | 1.76 (1.10-2.80) | **0.018** |
| MHO (n=286) | 46.3 |  | 0.59 (0.39-0.87) | **0.008** |  | 0.58 (0.39-0.87) | **0.008** |
| MUO (n=399) | 130.8 |  | 1.52 (1.11-2.09) | **0.010** |  | 1.56 (1.12-2.16) | **0.009** |
| CV death |  |  |  |  |  |  |  |
| MHNW (n=208) | 48.93 |  | Ref. |  |  | Ref. |  |
| MUNW (n=75) | 107.13 |  | 2.17 (1.25-3.77) | **0.006** |  | 2.33 (1.31-4.11) | **0.004** |
| MHO (n=286) | 23.64 |  | 0.50 (0.30-0.86) | **0.012** |  | 0.51 (0.30-0.88) | **0.015** |
| MUO (n=399) | 77.54 |  | 1.54 (1.02-2.32) | **0.040** |  | 1.59 (1.04-2.44) | **0.034** |
| Male and age ≥ 60 years | |  |  |  |  |  |  |
| All-cause death |  |  |  |  |  |  |  |
| MHNW (n=433) | 81.24 |  | Ref. |  |  | Ref. |  |
| MUNW (n=150) | 130.74 |  | 1.49 (1.06-2.09) | **0.020** |  | 1.50 (1.06-2.11) | **0.022** |
| MHO (n=564) | 51.47 |  | 0.64 (0.48-0.84) | **0.001** |  | 0.63 (0.48-0.83) | **0.001** |
| MUO (n=663) | 116.2 |  | 1.35 (1.06-1.70) | **0.013** |  | 1.36 (1.07-1.73) | **0.012** |
| CV death |  |  |  |  |  |  |  |
| MHNW (n=433) | 47.32 |  | Ref. |  |  | Ref. |  |
| MUNW (n=150) | 84.6 |  | 1.65 (1.08-2.53) | **0.022** |  | 1.67 (1.08-2.58) | **0.022** |
| MHO (n=564) | 32.23 |  | 0.68 (0.47-0.97) | **0.032** |  | 0.67 (0.47-0.96) | **0.028** |
| MUO (n=663) | 70.5 |  | 1.40 (1.03-1.89) | **0.031** |  | 1.40 (1.03-1.92) | **0.034** |
| Female and age < 60 years | |  |  |  |  |  |  |
| All-cause death |  |  |  |  |  |  |  |
| MHNW (n=91) | 146.26 |  | Ref. |  |  | Ref. |  |
| MUNW (n=44) | 145.07 |  | 0.95 (0.54-1.68) | 0.860 |  | 0.95 (0.52-1.74) | 0.875 |
| MHO (n=152) | 51.7 |  | 0.36 (0.22-0.59) | **<0.001** |  | 0.35 (0.21-0.58) | **<0.001** |
| MUO (n=216) | 79.93 |  | 0.50 (0.33-0.76) | **0.001** |  | 0.44 (0.28-0.69) | **<0.001** |
| CV death |  |  |  |  |  |  |  |
| MHNW (n=91) | 79.06 |  | Ref. |  |  | Ref. |  |
| **Table S3** (continued) | |  |  |  |  |  |  |
| **Subgroups** | **Incidence/**  **1000 person-y** |  | **Model 1** |  |  | **Model 2** |  |
|  |  |  | **HR (95% CI)** | ***P*-value** |  | **HR (95% CI)** | ***P*-value** |
| MUNW (n=44) | 88.65 |  | 1.04 (0.50-2.19) | 0.913 |  | 1.25 (0.57-2.75) | 0.584 |
| MHO (n=152) | 29.54 |  | 0.38 (0.20-0.74) | **0.004** |  | 0.38 (0.19-0.75) | **0.005** |
| MUO (n=216) | 36.46 |  | 0.43 (0.24-0.78) | **0.005** |  | 0.46 (0.25-0.86) | **0.015** |
| Female and age ≥ 60 years | |  |  |  |  |  |  |
| All-cause death |  |  |  |  |  |  |  |
| MHNW (n=270) | 68.84 |  | Ref. |  |  | Ref. |  |
| MUNW (n=92) | 156.95 |  | 1.97 (1.30-2.98) | **0.001** |  | 1.80 (1.17-2.76) | **0.007** |
| MHO (n=348) | 43.65 |  | 0.63 (0.43-0.92) | **0.017** |  | 0.62 (0.43-0.92) | **0.016** |
| MUO (n=447) | 130.8 |  | 1.72 (1.27-2.33) | **<0.001** |  | 1.59 (1.16-2.18) | **0.004** |
| CV death |  |  |  |  |  |  |  |
| MHNW (n=270) | 39.86 |  | Ref. |  |  | Ref. |  |
| MUNW (n=92) | 104.64 |  | 2.40 (1.42-4.05) | **0.001** |  | 2.31 (1.34-3.98) | **0.002** |
| MHO (n=348) | 21.83 |  | 0.55 (0.33-0.92) | **0.023** |  | 0.54 (0.33-0.92) | **0.022** |
| MUO (n=447) | 72.75 |  | 1.71 (1.15-2.56) | **0.008** |  | 1.66 (1.10-2.52) | **0.017** |

*HR* hazard ratio, *CI* confidence interval, *MHNW* metabolically healthy with normal weight, *MUNW* metabolically unhealthy with normal weight, *MHO* metabolically healthy with overweight or obesity, *MUO* metabolically unhealthy with overweight or obesity, *CV death* cardiovascular death. *P* values < 0.05 are presented in bold.

Model 1: adjusted for age, sex, smoking status, drinking status.

Model 2: adjusted for Model 1 + NYHA classification, LVEF, NT-proBNP, creatinine, LDL-C, previous MI, atrial fibrillation, COPD, past CABG, ACEI/ARB/ARNI, β-blocker, diuretics, SGLT2 inhibitors and other antidiabetic therapy.

**Table S4** HRs (95% CI) of primary outcomes based on the WHO definition of overweight and obesity

| **Subgroups** | **Incidence/**  **1000 person-y** |  | **Model 1** |  |  | **Model 2** |  |
| --- | --- | --- | --- | --- | --- | --- | --- |
|  |  |  | **HR (95% CI)** | ***P*-value** |  | **HR (95% CI)** | ***P*-value** |
| Overall |  |  |  |  |  |  |  |
| All-cause death |  |  |  |  |  |  |  |
| MHNW (n=1757) | 77.88 |  | Ref. |  |  | Ref. |  |
| MUNW (n=767) | 135.09 |  | 1.70 (1.46-1.97) | **<0.001** |  | 1.70 (1.45-1.98) | **<0.001** |
| MHO (n=1086) | 46.28 |  | 0.60 (0.51-0.72) | **<0.001** |  | 0.61 (0.51-0.73) | **<0.001** |
| MUO (n=1345) | 118.23 |  | 1.50 (1.31-1.71) | **<0.001** |  | 1.49 (1.30-1.70) | **<0.001** |
| CV death |  |  |  |  |  |  |  |
| MHNW (n=1757) | 46.32 |  | Ref. |  |  | Ref. |  |
| MUNW (n=767) | 87.62 |  | 1.86 (1.54-2.25) | **<0.001** |  | 1.87 (1.54-2.28) | **<0.001** |
| MHO (n=1086) | 23.67 |  | 0.52 (0.40-0.66) | **<0.001** |  | 0.52 (0.41-0.66) | **<0.001** |
| MUO (n=1345) | 67.53 |  | 1.45 (1.22-1.72) | **<0.001** |  | 1.46 (1.22-1.74) | **<0.001** |
| Male and age < 60 years | |  |  |  |  |  |  |
| All-cause death |  |  |  |  |  |  |  |
| MHNW (n=357) | 76.89 |  | Ref. |  |  | Ref. |  |
| MUNW (n=176) | 126.91 |  | 1.57 (1.14-2.17) | **0.006** |  | 1.62 (1.16-2.26) | **0.005** |
| MHO (n=241) | 48.2 |  | 0.64 (0.44-0.93) | **0.018** |  | 0.65 (0.45-0.94) | **0.021** |
| MUO (n=308) | 135.26 |  | 1.64 (1.24-2.17) | **<0.001** |  | 1.66 (1.24-2.22) | **<0.001** |
| CV death |  |  |  |  |  |  |  |
| MHNW (n=357) | 47.94 |  | Ref. |  |  | Ref. |  |
| MUNW (n=176) | 91.45 |  | 1.84 (1.24-2.73) | **0.002** |  | 1.87 (1.24-2.82) | **0.003** |
| MHO (n=241) | 22.95 |  | 0.49 (0.29-0.83) | **0.007** |  | 0.50 (0.30-0.84) | **0.008** |
| MUO (n=308) | 79.5 |  | 1.57 (1.10-2.24) | **0.013** |  | 1.54 (1.06-2.23) | **0.023** |
| Male and age ≥ 60 years | |  |  |  |  |  |  |
| All-cause death |  |  |  |  |  |  |  |
| MHNW (n=710) | 75.66 |  | Ref. |  |  | Ref. |  |
| MUNW (n=285) | 127.9 |  | 1.61 (1.26-2.08) | **<0.001** |  | 1.62 (1.25-2.10) | **<0.001** |
| MHO (n=469) | 48.06 |  | 0.65 (0.50-0.85) | **0.002** |  | 0.65 (0.49-0.86) | **0.002** |
| MUO (n=530) | 121.57 |  | 1.53 (1.24-1.89) | **<0.001** |  | 1.56 (1.26-1.94) | **<0.001** |
| CV death |  |  |  |  |  |  |  |
| MHNW (n=710) | 47.17 |  | Ref. |  |  | Ref. |  |
| MUNW (n=285) | 82.31 |  | 1.66 (1.21-2.28) | **0.002** |  | 1.61 (1.16-2.23) | **0.004** |
| MHO (n=469) | 26.49 |  | 0.58 (0.40-0.82) | **0.002** |  | 0.57 (0.39-0.82) | **0.002** |
| MUO (n=530) | 73.31 |  | 1.48 (1.13-1.93) | **0.004** |  | 1.51 (1.14-1.99) | **0.003** |
| Female and age < 60 years | |  |  |  |  |  |  |
| All-cause death |  |  |  |  |  |  |  |
| MHNW (n=187) | 106.56 |  | Ref. |  |  | Ref. |  |
| MUNW (n=98) | 153.51 |  | 1.34 (0.89-2.00) | 0.161 |  | 1.24 (0.81-1.91) | 0.322 |
| MHO (n=115) | 43 |  | 0.40 (0.23-0.67) | **<0.001** |  | 0.37 (0.22-0.63) | **<0.001** |
| MUO (n=170) | 67.09 |  | 0.57 (0.38-0.87) | **0.008** |  | 0.51 (0.33-0.78) | **0.002** |
| CV death |  |  |  |  |  |  |  |
| MHNW (n=187) | 59.6 |  | Ref. |  |  | Ref. |  |
| **Table S4** (continued) | |  |  |  |  |  |  |
| **Subgroups** | **Incidence/**  **1000 person-y** |  | **Model 1** |  |  | **Model 2** |  |
|  |  |  | **HR (95% CI)** | ***P*-value** |  | **HR (95% CI)** | ***P*-value** |
| MUNW (n=98) | 89.86 |  | 1.43 (0.84-2.43) | 0.190 |  | 1.52 (0.87-2.67) | 0.143 |
| MHO (n=115) | 21.5 |  | 0.35 (0.17-0.74) | **0.006** |  | 0.34 (0.16-0.72) | **0.005** |
| MUO (n=170) | 27.52 |  | 0.44 (0.24-0.80) | **0.007** |  | 0.43 (0.23-0.80) | **0.008** |
| Female and age ≥ 60 years | |  |  |  |  |  |  |
| All-cause death |  |  |  |  |  |  |  |
| MHNW (n=503) | 71.43 |  | Ref. |  |  | Ref. |  |
| MUNW (n=208) | 143.68 |  | 1.80 (1.35-2.40) | **<0.001** |  | 1.66 (1.23-2.25) | **<0.001** |
| MHO (n=261) | 42.72 |  | 0.60 (0.42-0.87) | **0.008** |  | 0.59 (0.41-0.86) | **0.005** |
| MUO (n=337) | 125.74 |  | 1.60 (1.24-2.08) | **<0.001** |  | 1.53 (1.17-2.00) | **0.002** |
| CV death |  |  |  |  |  |  |  |
| MHNW (n=503) | 39.26 |  | Ref. |  |  | Ref. |  |
| MUNW (n=208) | 90.22 |  | 2.15 (1.48-3.12) | **<0.001** |  | 2.09 (1.42-3.09) | **<0.001** |
| MHO (n=261) | 20.23 |  | 0.53 (0.31-0.89) | **0.017** |  | 0.51 (0.30-0.86) | **0.012** |
| MUO (n=337) | 69.64 |  | 1.68 (1.19-2.38) | **0.003** |  | 1.68 (1.17-2.42) | **0.005** |

*HR* hazard ratio, *CI* confidence interval, *WHO* World Health Organization, *MHNW* metabolically healthy with normal weight, *MUNW* metabolically unhealthy with normal weight, *MHO* metabolically healthy with overweight or obesity, *MUO* metabolically unhealthy with overweight or obesity, *CV death* cardiovascular death.

According to the WHO definition, overweight/obesity was classified as having a BMI of 25 kg/m² or higher, while obesity was classified as having a BMI of 30 kg/m² or higher. *P* values < 0.05 are presented in bold.

Model 1: adjusted for age, sex, smoking status, drinking status.

Model 2: adjusted for Model 1 + NYHA classification, LVEF, NT-proBNP, creatinine, LDL-C, previous MI, atrial fibrillation, COPD, past CABG, ACEI/ARB/ARNI, β-blocker, diuretics, SGLT2 inhibitors and other antidiabetic therapy.
